# Systematic review and meta-analysis of the management of acute uncomplicated diverticulitis: time to change traditional practice

**DOI:** 10.1007/s00384-024-04689-6

**Published:** 2024-07-17

**Authors:** Shafquat Zaman, Ali Yasen Mohamedahmed, Pradeep Thomas, Najam Husain

**Affiliations:** 1 Department of General Surgery, Russells Hall Hospital, Dudley Group NHS Trust, Dudley, West Midlands UK; 2 College of Medical and Dental Sciences, School of Medicine, University of Birmingham, Edgbaston, Birmingham, UK; 3 Department of General and Colorectal Surgery, Queen’s Hospital Burton, University Hospital of Derby and Burton NHS Trust, Derby, UK

Dear Editor,

We read with interest the commentary on our recently published article [1] in the *International Journal of Colorectal Disease* by Aamir et al. titled ‘Revisiting traditional practices in the management of acute uncomplicated diverticulitis: a call for change’ [2]. We appreciate their timely contribution and the important points raised.

The purpose of our original article was twofold: to evaluate outcomes of outpatient versus inpatient treatment and the use of antibiotics versus no antibiotics in the management of patients presenting with acute uncomplicated diverticulitis (Hinchey 1a).

Our conclusions, based on a mixture of observational and randomised trial data, were that a no-antibiotic approach in selected individuals is safe and feasible. Moreover, treating this cohort of patients in an outpatient setting is comparable to acute admission and in-hospital treatment (for outcomes including disease recurrence, treatment failure, and mortality).

The exact aetiology of diverticular disease and its associated complications remains unknown, but several risk factors may be involved. The incidence of disease increases with age, and other potential risk factors include genetics, a low-fibre diet, smoking, obesity, and a diet rich in red meat. The authors correctly identify potential confounding factors that may skew and affect our observations. They explain that specific risk factors causing diverticular disease will ultimately determine the course of the disease, its progression, and outcomes and call for risk stratification. In theory, this is a valid point and one which we agree with; however, in our view, it is impractical at present. In the UK and many developed nations, diverticular disease is very common and seen regularly on acute surgical takes. The idea of risk-stratifying patients, for instance, through genetic testing, is not currently feasible. Moreover, many other factors, including steroid use, immunosuppression, previous history of diverticular disease/diverticulitis, and co-morbidities (such as inflammatory bowel disease), will need to be taken into account. For this reason, sub-group analysis for included randomised trials alone was conducted to minimise confounders.

Moreover, the essence of conducting a systematic review and meta-analysis is to perform a synthesis of the available data. We are, therefore, limited by the quality of evidence published in the literature. We attempted to overcome some of these issues by performing analysis separately for groups including those with right-sided diverticulitis, patients presenting with a first-episode of disease flare-up, and RCTs alone.

The authors describe the selection of various antibiotics determined by disease severity (mild, moderate, severe) to treat patients. Appropriate antibiotic treatment is unlikely to be uniform across different geographical regions and will be dependent upon local antimicrobial resistance patterns, patient demographics, and microbiology input. The authors point to detailed antibiotic regimes from ‘guidelines’ published in a paper from 2004 [3] and suggest that these should be meticulously followed.

Emerging and more up-to-date evidence from well-designed, multi-centre trials over the last 10–15 years suggests that routine antibiotic use is unnecessary for acute uncomplicated diverticulitis [4–6]. Moreover, this approach is also supported by various guidelines, including the World Society of Emergency Surgery [7] and the National Institute for Health and Care Excellence (NICE) [8], which recommends adopting a no-antibiotic strategy in systemically well patients with uncomplicated disease.

Lastly, the authors suggest the use of serum marker CA-125 (mucin-16) to predict disease severity in patients presenting with acute diverticulitis. The basis of this is a recently published single-centre prospective observational study [9] involving 151 patients. Many other biomarkers, including CRP levels, may similarly be elevated and predict disease severity. In addition, CA-125 is not routinely performed, and its value as a prognostic biomarker warrants further investigation and validation. Moreover, this large glycoprotein is non-specific and elevated in many malignant (ovarian, endometrial, lung, breast, and gastrointestinal) and benign (uterine fibroids, pelvic inflammation) conditions, as well as menstruation and pregnancy.

In our study, patients were stratified on disease severity according to radiological findings (computed tomography scans). These scans are more accurate in determining the grade of disease (compared with currently available biomarkers), readily available, and often performed in patients presenting with acute abdominal symptoms.

In summary, our findings from the available literature support the reservation of antibacterial therapy for more complicated cases of acute diverticulitis. In addition, outpatient management is comparable to inpatient treatment in patients with uncomplicated acute diverticulitis. We acknowledge the potential for confounding variables affecting our conclusions and attempted to minimise these through sub-group analysis. We call for further well-designed, randomised, placebo-controlled trials to allow more definitive conclusions to be made.

## Data Availability

No datasets were generated or analysed during the current study.
